# Exome Sequencing and the Identification of New Genes and Shared Mechanisms in Polymicrogyria

**DOI:** 10.1001/jamaneurol.2023.2363

**Published:** 2023-07-24

**Authors:** Shyam K. Akula, Allen Y. Chen, Jennifer E. Neil, Diane D. Shao, Alisa Mo, Norma K. Hylton, Stephanie DiTroia, Vijay S. Ganesh, Richard S. Smith, Katherine O’Kane, Rebecca C. Yeh, Jack H. Marciano, Samantha Kirkham, Connor J. Kenny, Janet H. T. Song, Muna Al Saffar, Francisca Millan, David J. Harris, Andrea V. Murphy, Kara C. Klemp, Stephen R. Braddock, Harrison Brand, Isaac Wong, Michael E. Talkowski, Anne O’Donnell-Luria, Abbe Lai, Robert Sean Hill, Ganeshwaran H. Mochida, Ryan N. Doan, A. James Barkovich, Edward Yang, Dina Amrom, Eva Andermann, Annapurna Poduri, Christopher A. Walsh

**Affiliations:** 1 Division of Genetics and Genomics, Department of Pediatrics, Boston Children’s Hospital, and Allen Discovery Center for Human Brain Evolution, Boston, Massachusetts; 2 Division of Rheumatology, Hospital for Special Surgery, New York, New York; 3 Department of Neurology, Boston Children’s Hospital, Boston, Massachusetts; 4 Howard Hughes Medical Institute, Boston Children’s Hospital, Boston, Massachusetts; 5 Harvard-MIT MD/PhD Program, Harvard Medical School, Boston, Massachusetts; 6 Program in Medical and Population Genetics, Center for Genomic Medicine, Stanley Center for Psychiatric Research, The Broad Institute of MIT and Harvard, Cambridge, Massachusetts; 7 Department of Pharmacology, Feinberg School of Medicine, Northwestern University, Chicago, Illinois; 8 Department of Genetics and Genomics, United Arab Emirates University, United Arab Emirates; 9 GeneDx, Gaithersburg, Maryland; 10 Division of Medical Genetics, Our Lady of the Lake Health System, Baton Rouge, Louisiana; 11 Division of Medical Genetics, Department of Pediatrics Saint Louis University School of Medicine, St Louis, Missouri; 12 Department of Neurology, Harvard Medical School, Boston, Massachusetts; 13 Benioff Children’s Hospital, Departments of Radiology, Pediatrics, Neurology, and Neurological Surgery, University of California, San Francisco, San Francisco; 14 Department of Radiology, Boston Children’s Hospital, Boston, Massachusetts; 15 Neurogenetics Unit, Montreal Neurological Hospital and Institute, Montreal, Quebec, Canada; 16 Department of Neurology & Neurosurgery, McGill University, Montreal, Quebec, Canada; 17 Department of Neurology, Queen Fabiola Children’s University Hospital, Brussels, Belgium; 18 Pediatric Neurology Unit, Centre Hospitalier de Luxembourg, Grand-Duchy of Luxembourg; 19 Epilepsy Research Group, Montreal Neurological Hospital and Institute, Quebec, Canada; 20 Department of Human Genetics, McGill University, Montreal, Quebec, Canada

## Abstract

**Importance:**

Polymicrogyria is the most commonly diagnosed cortical malformation and is associated with neurodevelopmental sequelae including epilepsy, motor abnormalities, and cognitive deficits. Polymicrogyria frequently co-occurs with other brain malformations or as part of syndromic diseases. Past studies of polymicrogyria have defined heterogeneous genetic and nongenetic causes but have explained only a small fraction of cases.

**Objective:**

To survey germline genetic causes of polymicrogyria in a large cohort and to consider novel polymicrogyria gene associations.

**Design, Setting, and Participants:**

This genetic association study analyzed panel sequencing and exome sequencing of accrued DNA samples from a retrospective cohort of families with members with polymicrogyria. Samples were accrued over more than 20 years (1994 to 2020), and sequencing occurred in 2 stages: panel sequencing (June 2015 to January 2016) and whole-exome sequencing (September 2019 to March 2020). Individuals seen at multiple clinical sites for neurological complaints found to have polymicrogyria on neuroimaging, then referred to the research team by evaluating clinicians, were included in the study. Targeted next-generation sequencing and/or exome sequencing were performed on probands (and available parents and siblings) from 284 families with individuals who had isolated polymicrogyria or polymicrogyria as part of a clinical syndrome and no genetic diagnosis at time of referral from clinic, with sequencing from 275 families passing quality control.

**Main Outcomes and Measures:**

The number of families in whom genetic sequencing yielded a molecular diagnosis that explained the polymicrogyria in the family. Secondarily, the relative frequency of different genetic causes of polymicrogyria and whether specific genetic causes were associated with co-occurring head size changes were also analyzed.

**Results:**

In 32.7% (90 of 275) of polymicrogyria-affected families, genetic variants were identified that provided satisfactory molecular explanations. Known genes most frequently implicated by polymicrogyria-associated variants in this cohort were *PIK3R2, TUBB2B, COL4A1,* and *SCN3A*. Six candidate novel polymicrogyria genes were identified or confirmed: de novo missense variants in *PANX1*, *QRICH1*, and *SCN2A* and compound heterozygous variants in *TMEM161B*, *KIF26A,* and *MAN2C1,* each with consistent genotype-phenotype relationships in multiple families.

**Conclusions and Relevance:**

This study’s findings reveal a higher than previously recognized rate of identifiable genetic causes, specifically of channelopathies, in individuals with polymicrogyria and support the utility of exome sequencing for families affected with polymicrogyria.

## Key Points

**Question:**

In what proportion of individuals with polymicrogyria can a likely causal genetic variant be identified?

**Findings:**

In this genetic association study of 275 families with individuals affected by polymicrogyria, sequencing studies including exome sequencing were conducted that identified a likely causal genetic for polymicrogyria in 90 families (32.7% of sequenced families).

**Meaning:**

Broad, unbiased sequencing in individuals with polymicrogyria has a larger yield of genetic explanations than previously achieved through targeted sequencing and should be considered in the clinical setting.

## Teaser

This genetic association study assesses germline genetic causes of polymicrogyria in a large cohort and considers novel polymicrogyria gene associations.

## Introduction

Polymicrogyria is the most commonly identified malformation of cortical development (MCD), characterized by excessive folding of the cortex and typically accompanied by laminar disorganization.^1,2,3^ Individuals with polymicrogyria present in infancy or childhood with medically refractory epilepsy and are frequently diagnosed with intellectual disability and other neurological deficits depending on the location and extent of cortical involvement.^4^ Although the criterion standard for polymicrogyria diagnosis is pathologic examination, in clinical practice, polymicrogyria is typically identified on magnetic resonance imaging (MRI) during workup of epilepsy or developmental delay, where irregular gyration with scalloping of the gray-white matter junction is observed.^4^ Polymicrogyria can occur in isolation or with other brain malformations (including cobblestone malformation, schizencephaly, gray matter heterotopia, microcephaly or macrocephaly, and corpus callosum, brain stem, or cerebellar abnormalities) and can be part of multisystem syndromes.^5^

Nongenetic events (eg, in utero infections and ischemic insults) and genetic variants that disrupt cortical migration and postmigrational processes are known to cause polymicrogyria. Unlike other brain malformations, such as microcephaly or lissencephaly, whose genetic causes correspond to 1 or a small number of underlying mechanisms,^6^ genes responsible for polymicrogyria span diverse mechanisms. Variants in over 50 genes and several chromosome abnormalities implicate tubulin subunits, collagens, centrosome proteins, and components of the mTOR pathway in the pathogenesis of polymicrogyria.^7^ As of 2020, recommendations for the genetic workup of malformations of cortical development estimate a 20% diagnostic yield for polymicrogyria in a clinical setting.^8^ However, this likely underestimates the rate of identifiable genetic causes of polymicrogyria because prior efforts to catalog polymicrogyria-associated genes were limited by small case numbers, a focused set of considered etiologies, and/or use of targeted gene sequencing panels.^7,9,10,11^

We report the results of custom gene panel–targeted next-generation sequencing or exome sequencing (ES) in 275 families with individuals affected by polymicrogyria with no known genetic etiology. Our analysis identified a genetic etiology for polymicrogyria in 32.7% (90 of 275) of families, attributed to both single nucleotide variants and structural variants. In addition to detecting pathogenic variants in genes previously linked to polymicrogyria (eg,* ADGRG1/GPR56*, *SCN3A*, *TUBB2B*), we discovered 6 genes linked to polymicrogyria for the first time (*QRICH1*, *TMEM161B*, *PANX1*, *KIF26A*, *SCN2A*, and *MAN2C1*). Nearly 20% of polymicrogyria-associated variants in this cohort impact genes encoding ion-conducting proteins, suggesting channelopathies are a more common cause of polymicrogyria than previously appreciated.

In summary, our findings further define the landscape of genetic causes of polymicrogyria in the largest cohort published to date to our knowledge, highlighting for future investigation a heterogeneous set of disease mechanisms that converge on polymicrogyria. These results also reveal monogenetic causes of polymicrogyria to be more common than previously documented and support clinical ES of affected individuals.

## Methods

### Recruitment and Phenotyping

Our report follows the Strengthening the Reporting of Genetic Association Studies (STREGA) reporting guideline^12^ for describing a genetic association study as applicable to our design. Individuals with polymicrogyria without reportable genetic diagnoses after variable prior clinical genetic characterization (from none to negative clinical exome) were referred by collaborating clinicians or self-referred by families. Following provision of written informed consent, clinical data were collected and reviewed for phenotypes including seizures and head circumference, in utero complications, and family history by our team of neurologists, epileptologists, geneticists, and genetic counselors. Families were included in the cohort if affected individual(s) exhibited polymicrogyria or established polymicrogyria-related malformations (schizencephaly or dysgyria) on brain MRI (or computed tomography, rarely), as determined by at least 1 expert pediatric neuroradiologist (E.Y. or A.J.B.), using imaging findings defined as reflective of polymicrogyria.^3,4,5^ When brain imaging was not available for review, families were included if the referring clinician or outside radiology report clearly indicated the presence of polymicrogyria or established polymicrogyria-related malformations. This study was approved by responsible national and institutional committees on human subject research, including primarily Boston Children’s Hospital, Beth Israel Deaconess Medical Center, Montreal Neurological Hospital and Institute, and Queen Fabiola Children’s University Hospital, Brussels, Belgium. Samples were accrued over more than 20 years (1994 to 2020), and sequencing occurred in 2 stages: panel sequencing (June 2015 to January 2016) and whole-exome sequencing (September 2019 to March 2020).

### Sequencing and Variant Interpretation

**Figure 1.**
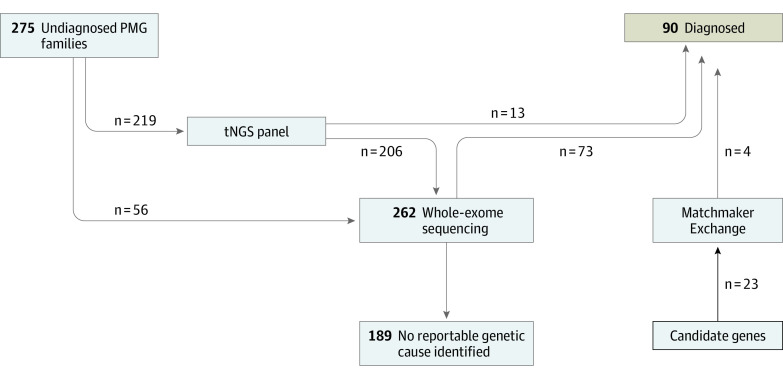
Workflow and Diagnostic Yield We performed panel sequencing, exome sequencing, or both on 275 families. After analysis for known genes associated with polymicrogyria (PMG), we also examined exomes for novel associations and used Matchmaker Exchange to build evidence for these associations. Our overall genetic explanation rate was 32.7% (90 of 275). tNGS indicates targeted next-generation sequencing.

DNA from affected individuals, as well as parents and unaffected siblings when available, was extracted from blood or other samples. Of 275 families in the study, 219 were initially assayed with a targeted next-generation sequencing panel comprising 155 manually curated genes (eTable 1 in Supplement 1) associated with polymicrogyria and MCDs that were amplicon sequenced as previously described.^13,14^ Families with explanatory variants identified on panel sequencing (n = 13) were considered solved and had no further sequencing after variant confirmation. A total of 262 families underwent ES for this study (Figure 1). ES and data processing were performed by the Genomics Platform at the Broad Institute of MIT and Harvard (eMethods in Supplement 1).

Evaluation of genetic variants identified by ES in the 262 families was performed independently by 2 or more investigators of the research team, including at least 1 licensed genetic counselor expert in research sequencing of individuals with MCDs (J.E.N. or A.L.). Most families were analyzed using *seqr*^15^ or NextCode; our parameters for variant filtration, prioritization, and interpretation are detailed in eMethods in Supplement 1.^16^ After initial review, variants of interest were presented for multidisciplinary group review, including neuroradiologists (E.Y. and A.J.B.), geneticists (D.H. and A.O.D.-L.), genetic counselors (J.E.N. and A.L.), neurologists (A.M., D.D.S., G.H.M., and C.A.W.), and neurogenetics research colleagues to prioritize candidates and discuss their classification alongside existing literature. Candidate variants were validated by Sanger sequencing in affected individuals and all available family samples to confirm segregation and variant phase or de novo status when possible. We confirmed nontrisomy CNVs identified on ES using droplet digital polymerase chain reaction or through clinical genetic sequencing.^17^

In 26 families, we identified candidate variants in 23 unique genes not previously associated with polymicrogyria. In an effort to identify consistent genotype-phenotype relationships for these genes, we used the Matchmaker Exchange (MME)^18^ platform to seek independent families with similar gene variants. We assessed potential matches for related phenotypes, mechanism of variant effect, and mode of inheritance.

### Head Size Association Analysis

Individuals affected with polymicrogyria were assigned to gene categories (nuclear, mTOR, ion conducting, signaling, cilia/centrosome, microtubule, collagen/vascular, and other) according to the polymicrogyria-associated gene containing their identified variant (eMethods and eResults in Supplement 1). In each category, we counted the number of individuals with or without (1) macrocephaly, (2) normocephaly, and (3) microcephaly. Macrocephaly was defined as head circumference greater than or equal to 2.5 SDs above the mean, normocephaly as head circumference between −2.5 and 2.5 SDs, and microcephaly as head circumference less than or equal to −2.5 SDs. For each head size range, we combined counts across the 8 gene module categories to produce an overall proportion. We used the 2-tailed Fisher exact test to compare the proportion of individuals with macrocephaly, normocephaly, or microcephaly in each gene moule category vs that in all individuals. We performed Fisher exact tests with the null hypothesis that the proportion of individuals with macrocephaly, normocephaly, or microcephaly in a module was the same as that in all individuals and adjusted results using the Benjamini-Hochberg method for multiple-testing correction. Two-sided *P* values were statistically significant at less than .05. Analysis took place between August and September 2022.

## Results

Figure 1 demonstrates the study organization, where samples from families with polymicrogyria underwent panel sequencing or ES to uncover their genetic etiologies. In total, we identified variants that explain 90 of 275 families (32.7%) with polymicrogyria, including single nucleotide variants in 86 families and copy number variants (CNVs) in 4 families (eTable 2 in Supplement 2). Concordant with prior literature,^2,7,8^ the most common genetic etiologies we found for polymicrogyria were in the *PIK3R2* (9 families) and *TUBB2B* (6 families) genes. We also identified 6 families with variants in the *COL4A1* or *COL4A2* gene. Beyond these established genetic causes, we were able to make confident genetic associations in several families with phenotypic similarities to recently reported syndromes, such as in 2 individuals with pathogenic variants in genes encoding AP4-complex proteins (*AP4E1* and *AP4M1*).^19^ Additional genetic causes of polymicrogyria we detected by ES include 3 pathogenic *PTEN* variants^20^ and a novel 3-base pair in-frame deletion in *MAST1*, consistent in variant class and individual phenotype with recent reports of a syndrome of mega-corpus-callosum syndrome with cerebellar hypoplasia and cortical malformations.^21^

### Novel Genetic Associations With Polymicrogyria

The Table summarizes novel polymicrogyria genes identified from our analyses. Using the MME platform, we recognized consistent genotype-phenotype relationships among multiple families with polymicrogyria for 4 genes not previously associated with human disease: *KIF26A*, *TMEM161B*, *PANX1*, and *MAN2C1.* Phenotypic expansions of 2 known disease genes to include polymicrogyria are supported by multiple families within our cohort: *SCN2A* (3 families), associated with developmental and epileptic encephalopathy, and *QRICH1* (4 families), associated with Ververi-Brady Syndrome. *SCN2A* was recently reported as potentially associated with polymicrogyria,^9^ and our findings solidify this association.

**Table.** Novel PMG-Associated Genes Identified in This Cohort Listed by Individual

Individual code	Gene	Transcript	Nucleotide change	Protein change	Zygosity, inheritance pattern	PMG distribution^a^	Head circumference, *z* score (age)
PMG14801	*KIF26A*	NM_015656.2	c.2845C>T, c.4676C>T	p.Pro949Ser, p.Ala1559Val	Compound heterozygous, AR	PMG (by report)	0.3 (30 y)
PMG20601	*PANX1*	NM_015368.4	c.40G>C	p.Asp14His	Heterozygous, AD de novo	Perisylvian PMG, extensive bilateral (R > L)	−4.2 (1 y, 1 mo)
PMGSL101	*PANX1*	NM_015368.4	c.110T>G	p.Met37Arg	Heterozygous, AD de novo	Extensive PMG bilateral (L > R) with closed lip schizencephaly in posterior left frontal lobe	−1.9 (6 y, 7 mo)
PMG24901	*PANX1*	NM_015368.4	c.1013A>C	p.Asn338Thr	Heterozygous, AD de novo	R-sided PMG and global reduced WM volume	Unknown (10 mo)
PMG11701	*QRICH1*	NM_198880.3	c.1150_1153del	p.Phe384GlnfsTer5	Heterozygous, AD de novo	Perisylvian PMG, bilateral, more severe posteriorly	−2.3 (3 y, 6 mo)
PS5201	*QRICH1*	NM_198880.3	c.304del	p.Val102PhefsTer144	Heterozygous, AD de novo	Perisylvian PMG, bilateral, parieto-occipital predominance	Unknown
BFP903-905	*TMEM161B*	NM_153354.5	c.580G>A	p.Glu194Lys	Compound heterozygous, AR	Diffuse PMG, bilateral (neuropathologic examination) of BFP906, 903-905 similar clinical syndrome with no imaging	Unknown
			c.362C>T	p.Thr121Ile			
PMG12601	*SCN2A*	NM_001040142.2	c.2548C>G	p.Arg850Gly	Heterozygous, AD de novo	Posterior perisylvian PMG, bilateral (R > L)	0.7 (15 mo)
PMG22701	*SCN2A*	NM_001040142.2	c.4919T>A	p.Ile1640Asn	Heterozygous, AD de novo	Posterior perisylvian PMG, bilateral	−0.7 (6 wk)
PMG19501	*SCN2A*	NM_001040142.2	c.1688G>A	p.Arg563His	Heterozygous	Cobblestone malformation, probable PMG (by report)	−4.3 (3 y, 5 mo)
PS4501	*MAN2C1*	NM_006715.4	c.2612G>C, c.601-2A>G	p.Cys871Ser	Compound heterozygous, AR	Perisylvian PMG, bilateral (R > L)	−1.6 (1 mo)

Abbreviations: AD, autosomal dominant; AR, autosomal recessive; L, left; PMG, polymicrogyria; R, right; WM, white matter.

^a^ Unless otherwise stated, magnetic resonance imaging was used.

#### PANX1

**Figure 2.**
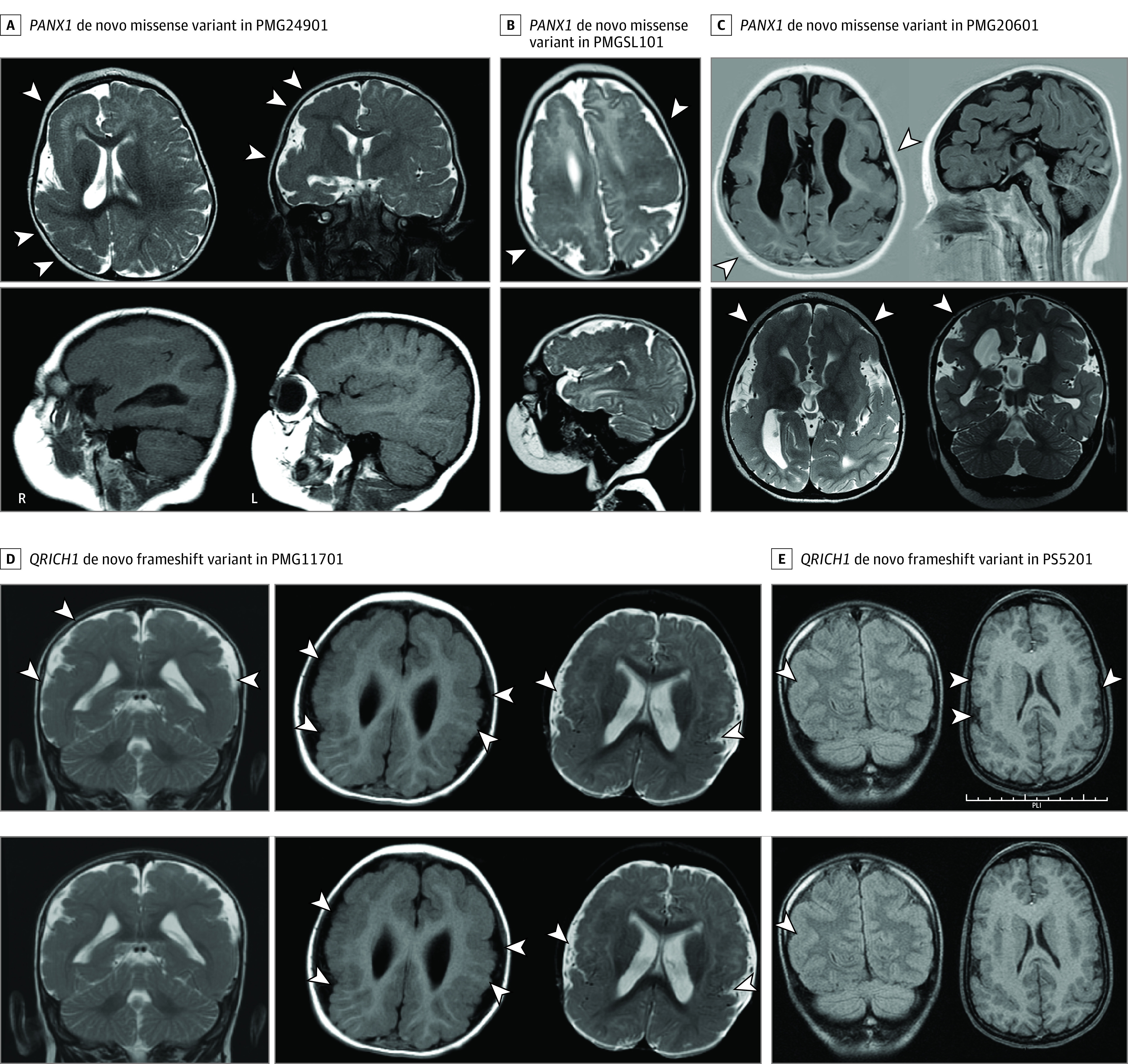
Magnetic Resonance Images From Affected Individuals With *PANX1* and *QRICH1* Variants A, PMG24901 has a de novo missense variant in *PANX1* (p.Asn338Thr) and unilateral right severe polymicrogyria and agyria with an unremarkable left hemisphere. B, PMGSL101 has a de novo missense variant in *PANX1* (p.Met37Arg) and has diffuse bilateral polymicrogyria with closed lip schizencephaly. C, PMG20601 has a de novo missense variant in *PANX1* (p.Asp14His) as well as diffuse bilateral polymicrogyria, worse on the right than left, and poor myelination. D, PMG11701 has a de novo frameshift variant in *QRICH1*, p.Phe384GlnfsTer5. E, PS5201 has a de novo frameshift variant in *QRICH1*, p.Val102PhefsTer144. Example regions of polymicrogyria are marked with white arrowheads.

We identified 3 individuals with candidate de novo missense variants in *PANX1* and extensive polymicrogyria on MRI (Figure 2A-C). PMG20601 presented with microcephaly and seizures. Imaging showed severe bilateral generalized polymicrogyria partially sparing the occipital cortices. PMGSL101 also had bilateral polymicrogyria and microcephaly with left-sided closed lip schizencephaly and no documented seizures. PMG24901 presented with unilateral severe right hemisphere polymicrogyria and left optic nerve hypoplasia but without apparent abnormality of the left cerebral hemisphere. PANX1, a member of the pannexin family of proteins, is highly expressed in cortical development.^22^ PANX1 subunits have been demonstrated to form homoheptameric single-membrane channels and play a role in calcium wave propagation,^23^ purinergic signaling,^24^ and *N*-methyl-d-aspartate current modulation.^25^ In animal models, Panx1 regulates neuronal cell proliferation and dendritic spine development.^26,27^

#### QRICH1

Two unrelated individuals with polymicrogyria affecting the perisylvian regions with parietal lobe predominance (PS5201 and PMG11701; Figure 2D and E) showed candidate de novo truncating variants in *QRICH1*. Although these cases displayed similarly affected brain regions, PMG11701 had more extensive polymicrogyria than PS5201. The parietal predominance in both individuals is notable because this polymicrogyria pattern is rare, accounting for less than 3% to 5% of polymicrogyria cases.^4^ Variants in *QRICH1* have been linked to Ververi-Brady syndrome, a rare condition of intellectual disability and mild dysmorphic features reported in only 38 individuals to date and to our knowledge; however, MRI findings beyond minor structural abnormalities have not been reported.^28,29,30,31,32^ QRICH1 was recently characterized as a transcription factor that is upregulated in response to endoplasmic reticulum stress and then activates a transcriptional module increasing protein flux into the endoplasmic reticulum.^33^ PMG11701 and PS5201 show dysmorphic features consistent with syndromes associated with *QRICH1* variants (eg, philtrum abnormality) and their shared uncommon parietal-predominant polymicrogyria suggests a common disease process. Thus, we report parietal predominant polymicrogyria as a phenotype associated with *QRICH1* variants.

Sequencing of our cohort identified polymicrogyria cases associated with de novo variants in *SCN2A,* as well as rare inherited variants in *TMEM161B, KIF26A*, and *MAN2C1*. Details of these associations have been recently reported (*TMEM161B*,^34^ *MAN2C1*,^35^ *KIF26A*,^36^ and *SCN2A*^9^).

### CNVs Associated With Polymicrogyria

Multiple CNV loci have been previously associated with syndromes that can exhibit polymicrogyria, although CNVs account for only 3% to 5% of genetically diagnosed cases in the literature.^37,38^ Accordingly, we found that polymicrogyria could be attributed to a CNV in 4.4% (4 of 90) of families with identified genetic explanations. These cases included two 22q microdeletions, one 6q deletion, and 1 previously undiagnosed trisomy 18 (eTable 2 in Supplement 2).

### Shared Mechanisms Among Polymicrogyria Genes

**Figure 3.**
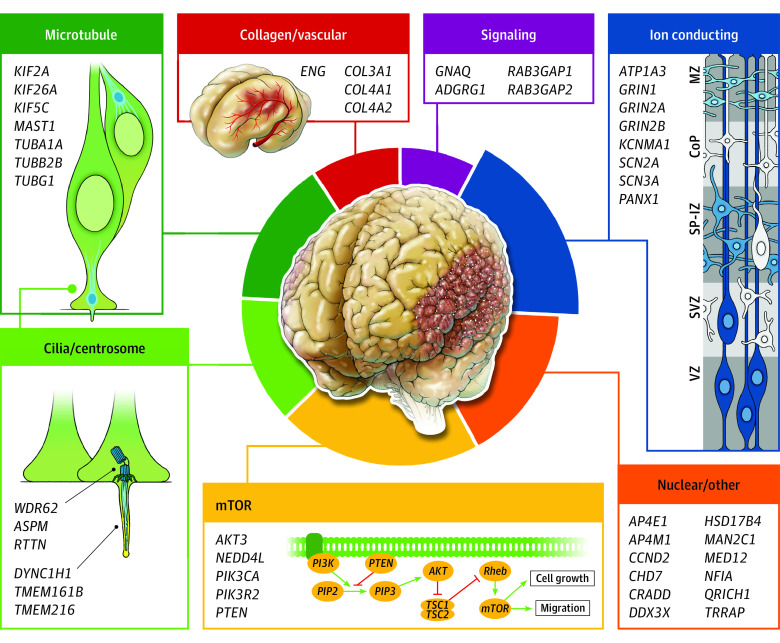
Summary of Genes Associated With Polymicrogyria by Category Relative proportion in each category of polymicrogyria-associated genes identified in this study is summarized. The largest category includes genes encoding mTOR pathway proteins, followed by those encoding ion-conducting proteins. CoP indicates cortical plate; MZ, marginal zone; SP-IZ, subplate intermediate zone; SVZ, subventricular zone; VZ, ventricular zone.

To organize the genetic causes of polymicrogyria according to biological categories, we generated a protein-protein interaction network map of proteins encoded by the polymicrogyria-associated genes identified in this cohort using the STRING database (eResults and eFigure in Supplement 1).^39^ We manually assigned genes without computer-assigned connections to modules through literature review. This network map reflects several previously recognized polymicrogyria-associated categories (tubulins, mTOR pathway) and defines new ones, such as ion-conducting proteins. The relative proportion in each category of polymicrogyria-associated genes identified in this study is summarized in Figure 3. Genes of the ion-conducting protein category, accounting for 17.7% (16 of 90) of identified genetic associations, composed the second largest category, behind the mTOR pathway module (19 of 90 [21.1%]) and ahead of the tubulin module (12 of 90 [13.3%]).

### Head Size in Individuals With Polymicrogyria

**Figure 4.**
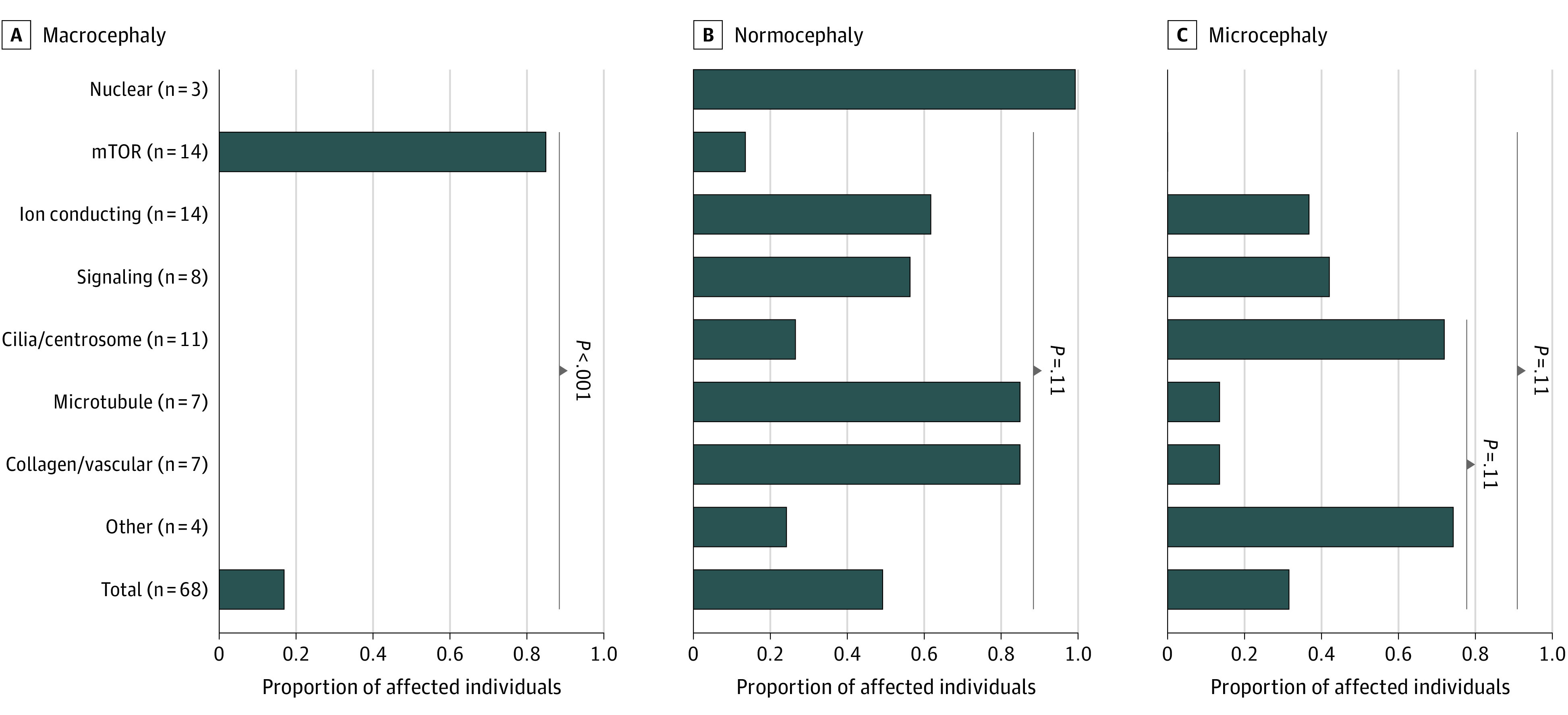
Head Size Associations With Polymicrogyria Categories Proportion of individuals affected with polymicrogyria that have macrocephaly, normocephaly, or microcephaly in each gene category, compared with that in all affected individuals. Shown are the Fisher exact test comparisons yielding the 4 smallest Benjamini-Hochberg–adjusted *P* values. Macrocephaly was associated with the mTOR category specifically, and microcephaly was absent in this category.

Polymicrogyria frequently co-occurs with either macrocephaly or microcephaly, and head size is easily assessed clinically. To test whether head size predicted polymicrogyria etiology, we analyzed the proportions of 68 affected individuals in whom genetic etiologies were determined that were microcephalic, normocephalic, or macrocephalic (Figure 4). Most individuals with polymicrogyria in our cohort either had normocephaly or microcephaly and had variants in genes from multiple categories. However, individuals with polymicrogyria and documented macrocephaly had variants only in genes encoding components of or related to the mTOR pathway (*PIK3R2, PI3KCA, AKT3,* and *PTEN*). Individuals with mTOR-related polymicrogyria also tended to be underrepresented in the microcephalic and normocephalic groups. This suggests that polymicrogyria co-occurring with macrocephaly should specifically prompt consideration of mTORopathies.

## Discussion

We identified explanatory genetic variants in 90 of 275 families (32.7%) in our cohort of individuals affected with polymicrogyria. Sequencing of this cohort contributed to first descriptions of several genes with novel or recent polymicrogyria associations (*TMEM161B*, *SCN2A*, *KIF26A*, *QRICH1*, *PANX1*, and *MAN2C1*), whether as phenotypic expansions of known neurological syndromes or as a syndrome with brain disease for the first time (*PANX1*). We provide a molecular categorization of genetic causes of polymicrogyria to accompany the phenotypic (clinical and imaging) descriptive categorizations already in use.^1,4^ The identification of *PANX1* and *SCN2A* variants in this cohort expands the set of ion-conducting proteins implicated in polymicrogyria. *QRICH1* is particularly interesting because it is one of only a few transcription factors implicated in polymicrogyria (along with *NFIA*,^40^ *EOMES/TBR2*,^41^ and *PAX6*,^42^ although none of these genes showed variants in our cohort). Finally, we note that the co-occurrence of polymicrogyria and macrocephaly should raise suspicion of gene variants that modulate the mTOR pathway.

We found genetic explanations for 32.7% of families with polymicrogyria primarily investigated by ES, evidence that the percent of polymicrogyria cases with genetic causes is higher than the approximately 20% diagnostic yield of clinical targeted gene panel sequencing.^7,8,9^ The higher rate of genetic explanation in our study is due to (1) the identification of multiple families with novel polymicrogyria-gene associations enabled by a large cohort and use of MME, (2) the predominant use of ES rather than a targeted gene panel, (3) the inclusion of CNV calling in our exome analysis that is not routinely performed clinically (although many patients will have had a prior chromosomal microarray), (4) the recent proliferation of documented genetic syndromes associated with polymicrogyria, and (5) the ascertainment of this cohort, on which analyses have been iteratively performed over many years as the literature has evolved. In several cases where we made associations based on previously published reports, despite polymicrogyria not being fully penetrant in syndromes associated with those genes (eg, *ENG, TRRAP*, *MED12*, 22q microdeletions), ES sequencing and candidate variant identification, coupled with rereview of clinical records, led to substantiation of their genetic explanations. We further demonstrate the utility of ES and relative limitation of commercial MCD panels by directly comparing the ability of various gene panels to detect the spectrum of findings that we identified across our cohort (eResults, eTable 3, and eDiscussion in Supplement 1). The families for which we did not find a genetic etiology could have rare variants in undiscovered monogenic polymicrogyria genes, somatic variants in known monogenic polymicrogyria genes, common variants of small effect size, congenital infections, or in utero ischemia.

The families recruited to our study had variable prior clinical and genetic characterization, from none to negative clinical ES. All cases were included if they were referred for research sequencing with no clear genetic explanation, regardless of severity or distribution of their polymicrogyria or co-occurring syndromic features. Thus, an even higher rate of monogenic or CNV causes of polymicrogyria would likely be detected if trio ES were applied prospectively in patients with polymicrogyria without prior testing. The frequent identification of variants in genes associated with known, named syndromes in several families, in a cohort where the key inclusion criterion for sequencing was polymicrogyria irrespective of other features, suggests that the presence of polymicrogyria in a patient is sufficient reason to perform broad sequencing, such as ES, for a family in search of a genetic diagnosis, with the possible exception of polymicrogyria that co-occurs with macrocephaly, in which case a targeted mTOR panel might precede ES.

Of 90 families in our polymicrogyria cohort with identified genetic explanations, 16 (17.7%) had variants in genes encoding ion-conducting proteins, on par with categories that have previously been considered the main genetic etiologies of polymicrogyria, such as mTORopathies and tubulinopathies. Traditionally associated with epilepsy in the absence of brain malformation, genes encoding ion-conducting proteins have been associated with polymicrogyria in multiple case series^43,44,45^; however, even recent cohort studies and reviews of polymicrogyria have discussed channelopathies as an exceptional or emerging, rather than common cause of polymicrogyria, and such genes are not included in most MCD gene panels.^1,2,3,8,9^ Many ion-conducting proteins are ubiquitously expressed in the developing brain with cell type and temporal specificity, which suggests that control of ionic flux is a key factor in normal cortical folding.^43^ Our study suggests that polymicrogyria should be considered part of the phenotypic spectrum of channelopathies, and sequencing of genes associated with ion-conduction should be included in genetic investigations of any individual with polymicrogyria.

### Strengths and Limitations

We study the largest sequenced polymicrogyria cohort to date to our knowledge and use an unbiased ES approach in addition to a gene panel sequencing approach. Our use of MME also enabled novel gene discovery to expand the set of genes associated with polymicrogyria. The major limitation of this study is that the cohort was ascertained over time and the analyses are retrospective, which limits the uniformity of imaging quality and in some cases the amount of clinical information available. However, in rare pediatric conditions, such as MCDs, especially polymicrogyria, which is widely heterogeneous in its scope and presence, a study like ours provides one of the only feasible ways to catalog large numbers of affected individuals effectively. Next, our sequencing depth precluded reliable assessment of mosaic variants, which may cause a fraction of polymicrogyria cases.^7^ Finally, while our study does not consider the cost-effectiveness of ES relative to other diagnostic strategies, other recent studies have already suggested that ES as first-line diagnostic test in infants suspected of monogenic disorders is clearly cost-effective.^46^

## Conclusions 

This study’s findings reveal a higher than previously recognized rate of identifiable genetic causes, specifically of channelopathies, in individuals with polymicrogyria and support the utility of exome sequencing for families affected with polymicrogyria.

## Notes

Supplement 1. eMethods eResults eDiscussion eTable 1. Genes Included in tNGS PMG Panel eTable 3. Brain Malformation Gene Panels Analyzed Compared to ES eFigure. STRING protein-protein interaction analysis groups PMG-associated genes into different modules eReferences

Supplement 2. eTable 2. PMG-Associated Variants Identified in Cohort

Supplement 3. Polymicrogyria Genetics Research Network nonauthor collaborators

Supplement 4. Data sharing statement
