# Supplementary material for: Exome Sequencing and the Identification of New Genes and Shared Mechanisms in Polymicrogyria
Source: JAMA Neurol. 2023 Jul 24;80(9):980–8. doi: 10.1001/jamaneurol.2023.2363 (PMC10366952; doi:10.1001/jamaneurol.2023.2363)
Supplement: Supplement 1. — eMethods eResults eDiscussion eTable 1. Genes Included in tNGS PMG Panel eTable 3. Brain Malformation Gene Panels Analyzed Compared to ES eFigure. STRING protein-protein interaction analysis groups PMG-associated genes into different modules eReferences [file jamaneurol-e232363-s001.pdf]

Supplemental Online Content

Akula SK, Chen AY, Neil JE, et al; Polymicrogyria Genetics Research Network. Exome sequencing and the identification of new genes and shared mechanisms in polymicrogyria. *JAMA Neurol*. Published online July 24, 2023.
doi:10.1001/jamaneurol.2023.2363

eMethods
eResults
eDiscussion
eTable 1. Genes Included in tNGS PMG Panel
eTable 3. Brain Malformation Gene Panels Analyzed Compared to ES
eFigure. STRING protein-protein interaction analysis groups PMG-associated genes into different modules
eReferences

This supplemental material has been provided by the authors to give readers additional information about their work.

© 2023 Akula SK et al. *JAMA Neurology.*

eMethods

Exome Sequencing and SNV Variant Annotation

Libraries from DNA samples (>250 ng of DNA, at >2 ng/ul) were created with an Illumina Nextera or Twist exome capture (~38 Mb target) and sequenced (150 bp paired reads) to cover >80% of exonic targets with minimum 20x and a mean target coverage of >100x. Sample identity quality assurance checks were performed on each sample. The ES data were de-multiplexed, and each sample's sequence data were aggregated into a single Picard BAM file. ES data were processed through a customized pipeline based on Picard, using base quality score recalibration and local realignment at known indels. The BWA aligner was used for mapping reads to the human genome build 38 (hg38). SNVs and insertions/deletions (indels) were jointly called across all samples using Genome Analysis Toolkit (GATK) HaplotypeCaller package version 3.5. Default filters were applied to SNV and indel calls using the GATK Variant Quality Score Recalibration (VQSR) approach. Annotation was performed using Variant Effect Predictor (VEP).

Copy Number Variant Detection

Copy-number variants (CNVs) were discovered from ES following GATK-gCNV best practices.[1] Briefly, read coverage was first calculated for each exome using GATK CollectReadCounts. Then, all samples were subdivided into batches determined using a principal components analysis (PCA) of sequencing read counts for gCNV model training and execution. After batching, one gCNV model was trained per batch using GATK GermlineCNVCaller on a subset of training samples, and the trained model was then applied to call CNVs for each sample per batch. Finally, all raw CNVs were aggregated across all batches and post-processed using quality- and frequency-based filtering to produce a final CNV callset. Lastly, the variant call set (SNVs and CNVs) was uploaded to *seqr*[2] for collaborative analysis.

Variant Interpretation

Guided by the principles of the American College of Medical Genetics and Genomics (ACMG)[3], we considered variants with no greater than 0.01 frequency for a recessive model or 0.0001 frequency for a dominant or *de novo* model using gnomAD v2.1[4] for general population reference and evaluated segregation within families as appropriate. We evaluated the predicted consequence of each variant meeting these frequency criteria for conservation of the relevant amino acid compared to multiple orthologs, *in silico* functional prediction algorithms (e.g., CADD score), predicted effect on splicing (if intronic) using Alamut Visual Plus and considered any existing associations with disease, such as reported in ClinVar, HGMD, or published clinical literature.

Diagnostic Yield Comparison to Commercial Gene Panels

To assess the difference in diagnostic yield between commercially available brain malformation panels and ES for PMG specifically, we calculated the putative diagnostic yields of several commercial brain malformation panels (GeneDx - 102 genes – comprehensive brain malformation panel August 2021, Invitae - 163 genes – test code 5506, August 2021, NHS/UK - 45 genes – version 2.148, and University of Chicago - 130 genes – test code 1130, August 2021) in our cohort *in silico*, not counting families in which we identified CNVs (n=4), or families in which we identified novel candidate PMG genes (n=9). The genes included in each panel are listed in Supplementary Table 4.

Network Visualization of PMG Genes

To visualize functional relationships between genes implicated in PMG, we loaded all genes associated with PMG in our patient cohort into stringApp, which uses the STRING database of known and predicted protein-protein interaction data to construct and visualize the protein-protein interaction network.[5] We then manually assigned functional modules based on this network and literature review.

© 2023 Akula SK et al. *JAMA Neurology.*

eResults

Clinical Gene Panel Comparisons

Panel sequencing for genetic characterization of MCDs is frequently used before ES in the clinical setting due to the expense and analytical challenges of ES, and because panels may capture most of the diagnostic yield for conditions that have relatively few genetic causes (e.g., lissencephaly, or holoprosencephaly). However, gene panels have substantially lower diagnostic yield for PMG relative to other MCDs due to the heterogeneity of PMG-associated genes and the ever-expanding list of implicated genes that are not always included on panels.[6] We assessed the performance of four commercially available gene panels used in evaluation of MCDs on our PMG cohort, excluding from this calculation families with identified pathogenic CNVs and families in whom we made novel PMG-gene associations, as those may not have been reported as such on a clinical ES. The diagnostic yield of ES in our cohort, excluding the noted families, would have been 76/262 (29.0%), representing 39 known PMG genes, which is substantially higher than the commercial gene panels, even before novel gene discovery or CNV analysis. All four of the commercially available panels (eTable 4) performed similarly:

MCD Gene Panel	Diagnostic Yield
Invitae	49/262 (18.7%)
University of Chicago	46/262 (17.5%)
GeneDx	43/262 (16.4%)
NHS/UK	35/262 (13.3%)

All analyzed panels performed in line with current guideline estimates of the yield of diagnostic panel sequencing studies in PMG (~15-20%).[6] Of the diagnoses made by ES that the examined panels would miss, many belonged to the category of ion conducting proteins (e.g., *ATP1A3*, *SCN3A*), accounting for a third to a half of missed diagnoses across the panels (Invitae – 13/27, University of Chicago – 11/30, GeneDx - 16/33, NHS/UK – 16/40).

STRING Analysis of PMG Associated Genes

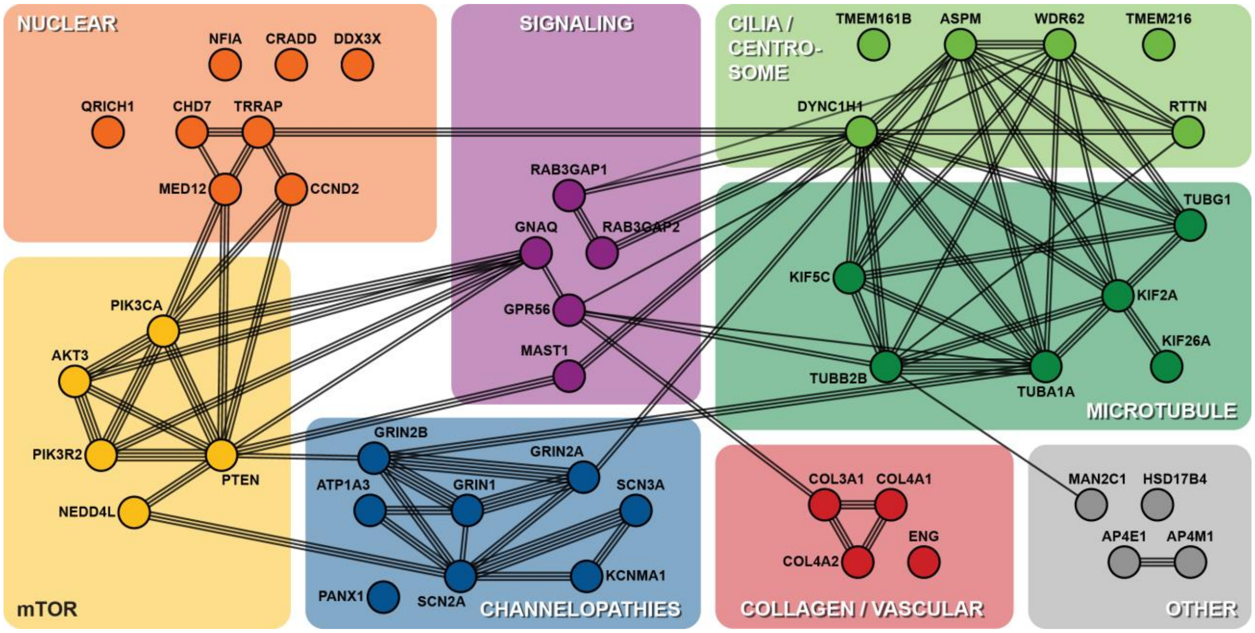

eFigure: STRING protein-protein interaction analysis groups PMG-associated genes into different modules. The number of lines denote the number of types of evidence for interaction, including reports in published literature, interactions reported in proteomic databases, and gene transcript co-expression.

© 2023 Akula SK et al. *JAMA Neurology.*

eDiscussion

Organizing PMG Etiologies

In this study, we searched for genetic etiologies for PMG in 275 families, identified several novel associations, and organized heterogenous genetic etiologies into a functional framework. Future genotype-phenotype studies of narrower groups, like excellent previous characterizations of specific groups of PMG patients with *PIK3R2* mutations[7] or tubulinopathies[8], will further delineate differences between the categories identified. Cohorts like this provide an invaluable resource to the clinical and research communities focused on the care of patients with MCD and those investigating the genetic underpinnings of brain development. Studies anchored in PMG genetics have not only demonstrated the necessity of genetic testing for diagnosis of MCDs but have also informed our understanding of brain development by uncovering roles for diverse cellular components, including growth signaling pathway enzymes, glycosylating enzymes, cytoskeletal components, and ion-conducting proteins.

PMG stands in contrast to other cortical malformations, in which relatively few genetic etiologies (if not mutations in few genes) cause most cases. For example, variants in just 17 genes, virtually all associated with microtubule binding proteins, account for 81% of all lissencephaly cases, with *LIS1* alone accounting for 40% of cases documented[9]. Also, more than half of primary microcephaly cases are caused by centrosome gene variants[10]. In contrast, genetic causes of PMG have been harder to catalogue because of their heterogeneity, and the overall diagnostic yield in PMG versus other MCDs will certainly be lower because PMG can result from non-genetic etiologies including *in utero* viral infections (estimated at 20-30% of cases)[6] and *in utero* ischemia.

Ion-conducting Proteins are a Common Cause of PMG

A recent excellent retrospective cohort study of PMG patients by Stutterd et al. used panel sequencing of 328 established or candidate brain malformation genes to estimate the frequency of germline mutations in 123 PMG patients, excluding CNVs, and determined a diagnostic yield of 20.3%.[11] The discrepancy in diagnostic yield between Stutterd et al.'s cohort (20.3%), and the rate of genetic explanations (not necessarily diagnostic yield) in our cohort (33%) is reasonably explained by the expansion of our sequencing breadth and ability for gene discovery; if we simply restricted our findings to the genes they included in their panel, our molecular explanation would have been similar to their reported yield. The genes that make up the yield difference are majority concentrated in a single category – genes encoding ion-conducting proteins. Further, the genes that make up that yield difference between previous studies or the gap between commercially available MCD panels and the yield of ES in our cohort are heavily concentrated in a single category – also genes encoding ion-conducting proteins. The mechanism by which ion channels influence cortical development is an active area of research[12], and may involve modulation of cell proliferation, migration, and differentiation.[13]

© 2023 Akula SK et al. *JAMA Neurology.*

eTable 1. Genes Included in tNGS PMG Panel

**Genes**			
AASS	*DROSHA*	*MAP2K2*	*RAD18*
ABCE1	*DSP*	*MCM2*	*RALGAPA1*
ACTN4	*DYNC1H1*	*MECP2*	*RB1*
AFF4	*EBF1*	*MLF1IP*	*RBL2*
AHI1	*EDNRB*	*MMP2*	*RTTN*
AKT3	*EGFR*	*MPL*	*SALL4*
ANXA1	*Eomes*	*MRE11A*	*SCN3A*
APOBEC3G	*EPM2AIP1*	*MSH6*	*SERBP1*
ARFGEF1	*FBLN2*	*MYC*	*SFI1*
ARHGAP12	*FBN1*	*MYCBP2*	*SHKBP1*
ARHGAP32	*FGD1*	*MYLK2*	*SKA3*
ARHGEF9	*FGF5*	*MYO9A*	*SMARCA2*
ARID1A	*FIG4*	*NBEA*	*SOX9*
ASPM	*FLNA*	*NCL*	*SRCAP*
ATP2B1	*FLNB*	*NCOR1*	*SRPX2*
B3GNT9	*FOXP2*	*NEDD1*	*STAG1*
BRCA2	*FRY*	*NF1*	*SUPT16H*
BTAF1	*GADD45A*	*NHEJ1*	*SUPT3H*
CBL	*GCK*	*NID1*	*SVIL*
CCND2	*GINS2*	*NRIP1*	*SYTL4*
CCP110	*GPR56*	*NSDHL*	*TACC3*
CDYL	*GPSM2*	*NUP160*	*TCF7L2*
CHD2	*HIST1H1C*	*OCLN*	*TNIK*
CITED2	*HIVEP2*	*OFD1*	*TOP2A*
COL12A1	*HSD17B4*	*PCDH12*	*TRRAP*
COL14A1	*IGSF1*	*PIK3CA*	*TTF2*
COL18A1	*ISPD*	*PIK3R2*	*TUBA1A*
COL3A1	*ITPR2*	*PLCG1*	*TUBA8*
COL5A1	*KALRN*	*PLCG2*	*TUBB*
COL6A3	*KIAA1279*	*POLA2*	*TUBB2B*
CORO1A	*KIF21B*	*POLE*	*TUBB3*
CSNK1A1	*KIF23*	*POU2F1*	*TUBG1*
CSNK1D	*KIF2A*	*PRPF8*	*TUBGCP3*
CTNND1	*KIF5C*	*PRRC2A*	*UNC13B*
DDX3X	*KNTC1*	*PSMB8*	*WDFY3*
DLL1	*KSR2*	*PTPN13*	*WDR62*
DMP1	*LAMA2*	*RAB18*	*ZEB1*
DNAI1	*LAMC3*	*RAB3GAP2*	*ZNRF3*
DOCK2	*LIG4*	*RAC3*	

© 2023 Akula SK et al. *JAMA Neurology.*

eTable 3. Brain Malformation Gene Panels Analyzed Compared to ES

**GeneDx Comprehensive Brain Malformations Panel**	**Invitae Brain Malformations Panel**	**NHS MCD Panel**	**UChicago MCD Panel**
ACTB	*ACTB*	*ACTB*	*ACTG1*
ACTG1	*ACTG1*	*ACTG1*	*ADGRG1*
ADGRG1	*ADGRG1*	*ADGRG1*	*AIMP1*
AHI1	*ADNP*	*AKT3*	*AKT3*
AKT3	*AHDC1*	*ARFGEF2*	*AMPD2*
AMPD2	*AKT3*	*ARX*	*APC2*
ARFGEF2	*AMPD2*	*CASK*	*ARFGEF2*
ARL13B	*APC2*	*CCND2*	*ARX*
ARX	*ARFGEF2*	*DCX*	*ASNS*
ASPM	*ARID1A*	*DYNC1H1*	*ASPM*
ATP6V0A2	*ARID1B*	*ERMARD*	*ATAD3A*
B3GALNT2	*ARX*	*FKRP*	*ATP6V0A2*
B9D1	*ASNS*	*FKTN*	*B3GALNT2*
B9D2	*ASPM*	*FLNA*	*B3GNT1*
C5orf42	*ATP6V0A2*	*GPSM2*	*B4GAT1*
CASK	*B3GALNT2*	*ISPD*	*BICD2*
CC2D2A	*B4GAT1*	*KIF1BP*	*BRF1*
CCND2	*BMP4*	*KIF2A*	*CASK*
CEP104	*C19orf12*	*KIF5C*	*CCND2*
CEP120	*CASK*	*LAMA2*	*CDK13*
CEP290	*CCM2*	*LAMB1*	*CDK5*
CEP41	*CCND2*	*LAMC3*	*CEP85L*
CHMP1A	*CDK13*	*LARGE*	*CHMP1A*
CIT	*CDK5*	*NDE1*	*CIT*
CSPP1	*CDON*	*OCLN*	*CLP1*
CUL4B	*CHMP1A*	*OPHN1*	*COASY*
DCHS1	*CIT*	*PAFAH1B1*	*COL18A1*
DCX	*CNOT1*	*PIK3CA*	*COL3A1*
DYNC1H1	*CNOT3*	*PIK3R2*	*COL4A1*
EXOSC3	*COASY*	*POMGNT1*	*CRADD*
FAT4	*COL18A1*	*POMT1*	*CRPPA*
FKRP	*COL3A1*	*POMT2*	*CUL4B*
FKTN	*COL4A1*	*RELN*	*DAG1*
FLNA	*COL4A2*	*RTTN*	*DCHS1*
GMPPB	*CP*	*SRPX2*	*DCX*
GPSM2	*CRADD*	*TMEM5*	*DMRTA2*
IFT172	*CUL4B*	*TUBA1A*	*DYNC1H1*
INPP5E	*DAG1*	*TUBA8*	*EML1*
ISPD	*DCHS1*	*TUBB*	*EXOSC3*
KATNB1	*DCX*	*TUBB2A*	*EXOSC8*
KIAA0586	*DIAPH1*	*TUBB2B*	*EXOSC9*
KIF1BP	*DISP1*	*TUBB3*	*FIG4*
KIF2A	*DLL1*	*TUBG1*	*FKRP*
KIF5C	*DMXL2*	*VLDLR*	*FKTN*
KIF7	*DPF2*	*WDR62*	*FLNA*
LAMB1	*DYNC1H1*		*GMPPB*
LAMC3	*EIF2AK2*		*GPR56*
LARGE1	*EMC1*		*GPSM2*

© 2023 Akula SK et al. *JAMA Neurology.*

**GeneDx Comprehensive Brain Malformations Panel**	**Invitae Brain Malformations Panel**		**UChicago MCD Panel**
MKS1	*EML1*		*GRIN2B*
NDE1	*ERMARD*		*KAT5*
NEDD4L	*EXOSC3*		*KATNB1*
NPHP1	*FA2H*		*KIF1BP*
NPHP3	*FAT4*		*KIF2A*
OCLN	*FGFR1*		*KIF5C*
OFD1	*FIG4*		*KIFBP*
OPHN1	*FKRP*		*LAMA2*
PAFAH1B1	*FKTN*		*LAMB1*
PIK3CA	*FLNA*		*LAMC3*
PIK3R2	*FOXA2*		*LARGE1*
POMGNT1	*FTL*		*LMNB1*
POMGNT2	*GAS1*		*MACF1*
POMK	*GLI2*		*MN1*
POMT1	*GMPPB*		*MTOR*
POMT2	*GPSM2*		*NDE1*
PQBP1	*GRIN2B*		*NEDD4L*
RAB18	*HIVEP2*		*OCLN*
RAB3GAP1	*IER3IP1*		*OFD1*
RAB3GAP2	*IQSEC2*		*OPHN1*
RARS2	*ISPD*		*OSGEP*
RELN	*KATNB1*		*PAFAH1B1 (LIS1)*
RPGRIP1L	*KCNMA1*		*PCLO*
RTTN	*KIF11*		*PEX1*
SEPSECS	*KIF1BP*		*PEX10*
SRD5A3	*KIF2A*		*PEX13*
SRPX2	*KIF7*		*PEX16*
TBC1D20	*KMT2E*		*PEX19*
TCTN1	*KRIT1*		*PEX2*
TCTN2	*L1CAM*		*PEX26*
TCTN3	*LAMA1*		*PEX3*
TMEM138	*LAMB1*		*PEX7*
TMEM216	*LAMC3*		*PHGDH*
TMEM231	*LARGE1*		*PI4KA*
TMEM237	*LRP2*		*PIK3R2*
TMEM5	*MACF1*		*POMGNT1*
TMEM67	*MED12*		*POMGNT2*
TSEN15	*MED17*		*POMK*
TSEN2	*MFSD2A*		*POMT1*
TSEN34	*MRE11*		*POMT2*
TSEN54	*NDE1*		*PPP1R12A*
TTC21B	*NEDD4L*		*PQBP1*
TUBA1A	*NPRL3*		*RAB11B*
TUBA8	*OCLN*		*RAB18*
TUBB	*OPHN1*		*RAB3GAP1*
TUBB2A	*PAFAH1B1*		*RAB3GAP2*
TUBB2B	*PANK2*		*RARS2*
TUBB3	*PDCD10*		*RELN*
TUBB4A	*PHGDH*		*RTTN*
TUBG1	*PIK3R2*		*SEPSECS*

© 2023 Akula SK et al. *JAMA Neurology.*

GeneDx Comprehensive Brain Malformations Panel	Invitae Brain Malformations Panel		UChicago MCD Panel
VLDLR	*PLA2G6*		*SHMT2*
VPS53	*PNKP*		*SLC25A46*
VRK1	*POMGNT1*		*SMPD4*
WDR62	*POMGNT2*		*SNAP29*
	POMK		*SON*
	POMT1		*SRD5A3*
	POMT2		*SRPX2*
	PPP1R12A		*TAF1C*
	PTCH1		*TBC1D20*
	RAB11B		*TBC1D23*
	RAB18		*TMEM5*
	RAB3GAP1		*TMTC3*
	RAB3GAP2		*TMX2*
	RAD21		*TOE1*
	RARS2		*TSEN15*
	RBM10		*TSEN2*
	RELN		*TSEN34*
	RERE		*TSEN54*
	RTTN		*TUBA1A*
	RXYLT1		*TUBA8*
	SEPSECS		*TUBB*
	SHH		*TUBB2A*
	SIN3A		*TUBB2B*
	SIX3		*TUBB3*
	SLC25A19		*TUBB4A*
	SMARCA4		*TUBG1*
	SMARCB1		*TUBGCP2*
	SMARCE1		*VLDLR*
	SMC1A		*VPS51*
	SNAP29		*VPS53*
	SON		*VRK1*
	SOX2		*WDR62*
	SRD5A3		
	STAG2		
	STAMBP		
	STIL		
	TBC1D20		
	TGIF1		
	TMTC3		
	TOE1		
	TRRAP		
	TSEN2		
	TSEN34		
	TSEN54		
	TUBA1A		
	TUBA8		
	TUBB2A		
	TUBB2B		
	TUBB3		
	TUBB4A		

© 2023 Akula SK et al. *JAMA Neurology*.

	**Invitae Brain Malformations Panel**		
	TUBG1		
	TUBGCP6		
	UBE3B		
	USP7		
	USP9X		
	VLDLR		
	VPS13A		
	VRK1		
	WDR45		
	WDR62		
	YWHAE		
	ZBTB18		
	ZBTB20		
	ZIC2		
	ZMIZ1		

© 2023 Akula SK et al. *JAMA Neurology*.

eReferences

1. Gabrielaite M, Torp MH, Rasmussen MS, et al. A Comparison of Tools for Copy-Number Variation Detection in Germline Whole Exome and Whole Genome Sequencing Data. *Cancers*. 2021;13(24):6283. doi:10.3390/cancers13246283

2. Pais LS, Snow H, Weisburd B, et al. Seqr *: A Web-Based Analysis and Collaboration Tool for Rare Disease Genomics*. Genetic and Genomic Medicine; 2021. doi:10.1101/2021.10.27.21265326

3. Richards S, Aziz N, Bale S, et al. Standards and guidelines for the interpretation of sequence variants: a joint consensus recommendation of the American College of Medical Genetics and Genomics and the Association for Molecular Pathology. *Genetics in Medicine*. 2015;17(5):405-424. doi:10.1038/gim.2015.30

4. Karczewski KJ, Francioli LC, Tiao G, et al. The mutational constraint spectrum quantified from variation in 141,456 humans. *Nature*. 2020;581(7809):434-443. doi:10.1038/s41586-020-2308-7

5. Szklarczyk D, Gable AL, Nastou KC, et al. The STRING database in 2021: customizable protein-protein networks, and functional characterization of user-uploaded gene/measurement sets. *Nucleic Acids Res*. 2021;49(D1):D605-D612. doi:10.1093/nar/gkaa1074

6. Oegema R, Barakat TS, Wilke M, et al. International consensus recommendations on the diagnostic work-up for malformations of cortical development. *Nat Rev Neurol*. 2020;16(11):618-635. doi:10.1038/s41582-020-0395-6

7. Mirzaa GM, Conti V, Timms AE, et al. Characterisation of mutations of the phosphoinositide-3-kinase regulatory subunit, PIK3R2, in perisylvian polymicrogyria: a next-generation sequencing study. *Lancet Neurol*. 2015;14(12):1182-1195. doi:10.1016/S1474-4422(15)00278-1

8. Romaniello R, Arrigoni F, Fry AE, et al. Tubulin genes and malformations of cortical development. *Eur J Med Genet*. 2018;61(12):744-754. doi:10.1016/j.ejmg.2018.07.012

9. Di Donato N, Timms AE, Aldinger KA, et al. Analysis of 17 genes detects mutations in 81% of 811 patients with lissencephaly. *Genet Med*. 2018;20(11):1354-1364. doi:10.1038/gim.2018.8

10. Jayaraman D, Bae BI, Walsh CA. The Genetics of Primary Microcephaly. *Annu Rev Genom Hum Genet*. 2018;19(1):177-200. doi:10.1146/annurev-genom-083117-021441

11. Stutterd CA, Brock S, Stouffs K, et al. Genetic heterogeneity of polymicrogyria: study of 123 patients using deep sequencing. *Brain Commun*. 2020;3(1). doi:10.1093/braincomms/fcaa221

12. Smith RS, Walsh CA. Ion Channel Functions in Early Brain Development. *Trends in Neurosciences*. 2020;43(2):103-114. doi:10.1016/j.tins.2019.12.004

13. Bates E. Ion Channels in Development and Cancer. *Annu Rev Cell Dev Biol*. 2015;31(1):231-247. doi:10.1146/annurev-cellbio-100814-125338

© 2023 Akula SK et al. *JAMA Neurology.*
