# Supplementary material for: Exome Sequencing and the Identification of New Genes and Shared Mechanisms in Polymicrogyria
Source: JAMA Neurol. 2023 Jul 24;80(9):980–8. doi: 10.1001/jamaneurol.2023.2363 (PMC10366952; doi:10.1001/jamaneurol.2023.2363)
Supplement: Supplement 3. — Polymicrogyria Genetics Research Network nonauthor collaborators [file jamaneurol-e232363-s003.pdf]

Supplemental Online Content: Nonauthor Collaborators
*First name, last name, and suffix (if applicable) are required and will appear in PubMed.

***Group Name(s): Polymicrogyria Genetics Research Network**							
***First Name and Middle Initial(s)**	***Last Name**	***Suffix (eg, Jr, III)**	Academic Degrees	Institution	Location (city, state/province, country)	Role or Contribution, eg, chair, principal investigator	Group (if more than 1 Group listed in the byline) and/or Subgroup (eg, Steering Committee)
Bassam	Abu-Libdeh						
Lihadh	Al-Gazali						
Muna	Al Saffar						
Edith	Alva Moncayo						
Dina	Amrom						
Eva	Anderman						
Anna-Kaisa	Anttonen						
Saunder	Barnes						
Sara	Barnett						
Todd	Barron						
Brenda J.	Barry						
Lina	Basel-Vanagaite						
Laila	Bastaki						
Luis	Bello-Espinosa						
Tawfeg	Ben-Omran						
Matthew	Bernard						
Carsten G.	Bonneman						
Blaise	Bourgeois						
Stephen	Brown						
Roberto H.	Caraballo						
Gergory	Cascino						
Michael	Clarke						
Monika	Cohen						
Yanick	Crow						
Bernard	Dan						
Kira A.	Dies						
William B.	Dobyns						
François	Dubeau						

Supplemental Online Content: Nonauthor Collaborators
*First name, last name, and suffix (if applicable) are required and will appear in PubMed.

***First Name and Middle Initial(s)**	***Last Name**	***Suffix (eg, Jr, III)**	Academic Degrees	Institution	Location (city, state/province, country)	Role or Contribution, eg, chair, principal investigator	Group (if more than 1 Group listed in the byline) and/or Subgroup (eg, Steering Committee)
Christelle	El Achkar						
Gregory M.	Enns						
Laurence	Faivre						
Laura	Flores-Sarnat						
John	Gaitanis						
Kuchukhidze	Giorgi						
Andrew	Green						
Alan	Guberman						
Renzo	Guerrini						
Micheil	Innes						
Richard	Jacobsen						
Sebastian	Jacquemont						
Samir	Khalil						
Joerg	Klepper						
Dimitri	Kranic						
Kalpathy	Krishnamoorthy						
Anna-Elina	Lehesjoki						
Dorit	Lev						
Richard J.	Leventer						
Emily	Lisi						
Valerie	Loik Ramey						
Sally Ann	Lynch						
Laila	Mahmoud						
David	Manchester						
David	Mandelbaum						
Daphna	Marom						
Deborah	Marsden						
Mayra	Martinez Ojeda						
Amira	Masri						

Supplemental Online Content: Nonauthor Collaborators
*First name, last name, and suffix (if applicable) are required and will appear in PubMed.

***First Name and Middle Initial(s)**	***Last Name**	***Suffix (eg, Jr, III)**	Academic Degrees	Institution	Location (city, state/province, country)	Role or Contribution, eg, chair, principal investigator	Group (if more than 1 Group listed in the byline) and/or Subgroup (eg, Steering Committee)
Livija	Medne						
Denis	Melanson						
David T.	Miller						
Anna	Minster						
Edward	Neilan						
Dang Khoa	Nguyen						
Heather E.	Olson						
Ignacio	Pascual-Castroviejo						
Philip L.	Pearl						
Daniela	Pilz						
Nada	Quercia						
Salmo	Raskin						
Miriam	Regev						
Lance	Rodan						
Cynthia	Rooney						
Michael	Rutlin						
Mustafa	Sahin						
Mustafa A.	Salih						
Pierre	Sarda						
Harvey B.	Sarnat						
Ingrid	Scheffer						
Joseph	Shieh						
Sharon E.	Smith						
Janet S.	Soul						
Siddharth	Srivastava						
Laszlo	Sztriha						
Donatella	Tampieri						
John	Tolmie						
Meral	Topçu						
Eugen	Trinka						

Supplemental Online Content: Nonauthor Collaborators
*First name, last name, and suffix (if applicable) are required and will appear in PubMed.

***First Name and Middle Initial(s)**	***Last Name**	***Suffix (eg, Jr, III)**	Academic Degrees	Institution	Location (city, state/province, country)	Role or Contribution, eg, chair, principal investigator	Group (if more than 1 Group listed in the byline) and/or Subgroup (eg, Steering Committee)
John	Tsai						
Jack	Tsao						
Sheila	Unger						
Iris	Unterberger						
Goekhan	Uyanik						
Kette	Valente						
Thomas	Voit						
Louise	Wilson						
Grace	Yoon						
