# Supplementary material for: Exome Sequencing and the Identification of New Genes and Shared Mechanisms in Polymicrogyria
Source: JAMA Neurol. 2023 Jul 24;80(9):980–8. doi: 10.1001/jamaneurol.2023.2363 (PMC10366952; doi:10.1001/jamaneurol.2023.2363)
Supplement: Supplement 4. — Data sharing statement [file jamaneurol-e232363-s004.pdf]

Data Sharing Statement

Akula. Exome Sequencing and the Identification of New Genes and Shared Mechanisms in Polymicrogyria. *JAMA Neurol*. Published July 24, 2023. doi:10.1001/jamaneurol.2023.2363

Data
Data available: Yes
Data types: Deidentified participant data
How to access data: Supplement contains all relevant data.
When available: With publication

Supporting Documents
Document types: None

Additional Information
Who can access the data: Anyone accessing the manuscript
Types of analyses: Any purpose
Mechanisms of data availability: In Supplement.
Any additional restrictions: N/A
